# Modelling the Effect of Age, Semester of Study and Its Interaction on Self-Reflection of Competencies in Medical Students

**DOI:** 10.3390/ijerph19159579

**Published:** 2022-08-04

**Authors:** Jannis Achenbach, Thorsten Schäfer

**Affiliations:** Center for Medical Education, Ruhr-University Bochum, 44801 Bochum, Germany

**Keywords:** self-reflection, peer-assisted learning, skills labs, self-assessment, competency-based learning

## Abstract

**Objectives:** Accurate self-assessment and -reflection of competencies are crucial skills for all health professions. The National Competence-Based Learning Objectives Catalogue (NKLM) guiding medical faculties in Germany points out reflection as a non-technical skill and competency-based medical education (CBME) as important approaches. In this context, the role and structure of curricula and skills labs evolved. Especially in peer-assisted trainings, reflection of competencies is important to improve self-regulated learning. Traditionally, we assume self-reflection skills to evolve automatically with learners’ experience. This approach aims to find empirical evidence for this assumption and implements self-reflection of competencies in clinical skills education. Here, we quantify the influence of age and semester of study and its interaction on the concordant self-reflection of students’ own competencies. **Methods:** Investigation was based on a retrospective analysis of evaluation data from peer-assisted “first aid” and “physical examination” courses in the skills labs of the medical faculty at the Ruhr-University Bochum, Germany. Participants were asked for self-assessed competencies before (*pre*) and after (*post*) the course. Additionally, they were asked to retrospectively re-rate their “before” competencies after completing the course (*post-pre*). Differences between *pre* and *post-pre* competencies were assessed as the concordant self-reflection in a moderated regression analysis. Group means and standard deviation were depicted using univariate analysis of variance (ANOVA) with post-hoc Tukey HSD testing in *IBM SPSS Statistics V.28*. Moderated regression and simple slope analyses were conducted to calculate interaction effects of age and semester of study on the concordant self-reflection. **Results:** As expected, participants (*n* = 168) showed significant progress in subjective self-assessment (*pre* vs. *post*) in all 18 assessed domains in the course (all *p* < 0.001). Additionally, participants self-assessed their previous competencies after the course (*post*-*pre*) differently than before the course (*pre)* in 11 out of 18 domains. Hereby, the interaction of age and semester of study explained a significant part of variance in the first aid course (∆*R*^2^ = 0.008, ∆*F* (1;1020) = 8.53, *p* < 0.005) and in the physical examination course (Δ*R*^2^ = 0.03, Δ*F* (1;10,280) = 10.72, *p* < 0.001). **Conclusions:** We quantified that interaction of age and semester has a significant influence on concordant self-reflection skills using a moderated regression analysis. Assumed as an indicator, we conclude that advanced and older students show less differences in *pre-* vs. *post-pre*-ratings. This has implications for curriculum development, postulating that an exposure to self-reflection as a metacognitive process should be introduced early in order to train competencies in health professionals. Prospective studies with competency-based assessments are necessary to validate findings.

## 1. Introduction

Self-reflection is defined as a complex metacognitive process and the capacity to consciously reflect on one’s own abilities [1,2,3]. It is influenced by personal experiences, individual behaviors, social interactions, and underlying neurobiological factors. It correlates with and is relevant to applying learning strategies [1,2]. Accurate self-assessment and reflection of one’s own competencies are furthermore important skills in medical professions in terms of learning effectiveness and patient safety. The ability to categorize one’s own competencies in an overall context was postulated to have a meaningful impact on self-corrective behaviors going along with improved professional skills in medical education [3,4]. Self-monitoring accuracy was therewith stated to enhance learning processes and have the potential for improving medical care [5]. This ability for self-reflection and critical thinking has been assessed in health professions, revealing that professional education has an impact on critical thinking [6]. Manifold approaches have focused on influences on self-reflection, critical reflection of competencies, and on learning processes in clinical simulation-based medical education [7,8,9,10,11]. Especially in the absence of the professional teacher, an ability for accurate self-assessment is fundamental in skill training; however, research has found that multiple influencing factors are unknown, revealing an urgent need to assess predictors and modulating factors [12]. This need has also been addressed by Blanch-Hartigan by performing meta-analyses and postulating that investigations are necessary to identify moderators that influence the self-assessment accuracy in medical students [13].

In view of the current National Competence-Based Learning Objectives Catalogue (NKLM), describing competencies and learning objectives as a guideline for German faculties and therewith for curriculum development, competency-based medical education (CBME) has become a main approach in medical education and teaching methods [14,15]. Because self-reflection is described within the NKLM as an integrative part of the medical curricula, recently, Schrempf et al. presented approaches concerning how to integrate this fundamental and important tool within the educational practice [16].

With regard to more practical-orientated curriculums, CBME, and practical teaching concepts, simulation and skills labs have been developed during the last decades [17,18]. The concept of peer-assisted learning (PAL) as a valuable didactic concept was developed to expand practical training in curricular as well as extracurricular, facultative simulation-based education of medical competencies [19]. Especially in skills training and PAL, reflection of students’ own competencies to improve self-regulated learning is important. Nevertheless, comprehensive implications and findings of influencing and modifiable factors are lacking until now, proclaiming a “need for predictive cues” [20].

**Figure 1:**
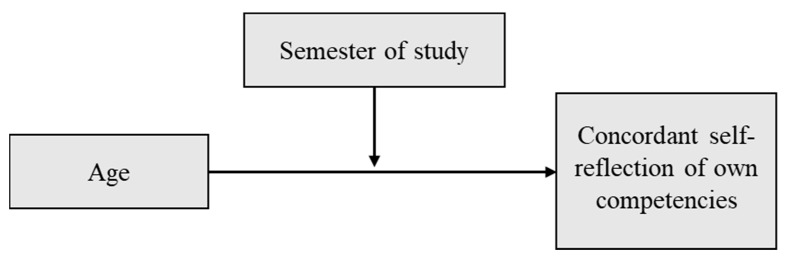
Hypothesized model of the conducted moderated regression analysis.

In this context, there is a research gap between the traditional assumption that self-reflection skills evolve automatically with learners’ experience and missing empirical evidence. As the main focus, our research therefore set out to analyze the hypothesized model of the influence of age and semester of study and their interaction on the concordant self-reflection of students’ self-assessed competencies by analyzing evaluation data (Figure 1). The further objective was to assess the perceived gain of self-reflected competencies with regard to practical contents of students attending PAL courses in skills labs and to implement self-reflection as a non-technical skill in clinical education.

## 2. Methods

### 2.1. Assessed Evaluation Data

Investigation was based on the retrospective analysis of evaluation data coming from peer-assisted courses in the skills labs of the Ruhr-University Bochum from three consecutive semesters (winter semester 2018/19, summer semester 2019, and winter semester 2019/2020). Data were analyzed from the “first aid” and “physical examination course”. The standardized, didactical training concept for peer tutors, course concepts, and the evaluation paradigm was trained as published earlier [21,22]. Tutors were trained in standardized didactic trainings and specialist trainings by medical experts. Structured module manuals were provided for training.

Competencies were self-assessed before (1: “*pre*”), after (2: “*post*”), and after the course with an imaginary-retrospective assessment of competencies before participating in the course (3: “*post-pre*”). Differences found with an alignment of “*pre*” and “*post-pre*” evaluations were assessed as the “*concordant self-reflection*” and implemented within the moderated regression analysis. The concordant self-reflection was therewith defined as the post-hoc correction in the self-assessment.

The evaluation of the “first aid” course included a survey of eight items (emergency call, basic resuscitation, use of an automated external defibrillator (AED), stable side position, bleedings, burning, helmet removal, complex first aid) to ask attendees for their own confidence in procedural handling of practical skills. Evaluation of the “physical examination course” asked for ten course-specific domains (medical history, hygiene, first patient impression, lungs-, cardiac-, abdominal-, neurologic-, orthopedic-, lymph nodes-, and thyroid gland examination). Self-assessment was documented using a six-point Likert scale differentiating between “strongly agree (=1)” and “strongly disagree (=6)” as the most severe expressions. All course attendees voluntarily took part in the evaluation concept, and data were assessed pseudonymized. Evaluation data were assessed as part of the routine course assessments; analysis and collection were conducted anonymously. Data were collected from twelve first aid and eight physical examination courses.

### 2.2. Statistical Analysis

Group means and standard deviation of the evaluation data were assessed using univariate analysis of variance (ANOVA) with post-hoc Tukey HSD testing for pair-wise analysis of different estimates on the self-assessment of competencies in IBM SPSS Statistics V.28. Adjustment for multiple testing was applied using Bonferroni corrections. Dependent variables were tested for normal distribution using the Kolmogorov–Smirnov test (data not shown). Homogeneity of variances was asserted using Levene’s Test. Detecting unequal variances, values were reported with Welch’s test. Chi-square tests were used for analyses of categorical and descriptive variables. Afterwards, moderated regression analysis was conducted to analyze interaction effects of age and semester of study on the concordant self-reflection of competencies. Simple slope analyses were calculated to illustrate effects.

## 3. Results

### 3.1. Peer-Assisted Learning Courses and Attendees

We retrospectively assessed evaluation data coming from three subsequent study semesters revealing an overall cohort of *N* = 169 participants within the first aid and physical examination course concept. To evaluate the peer-assisted extracurricular self-reflection data, we used data coming from the first aid (*n* = 129) and physical examination course (*n* = 40) with regard to modelling effects of age, semester of study, and their interaction on the congruent ability for self-reflection.

Course attendees’ demographics revealed 38.5% of the participants were in semester three, followed by 17.8% in semester two and 16.0% in semester one of their medical studies. The largest proportion of participants was 20 years old (21.3%), followed by 20.1% at the age of nineteen and 13% at the age of 21 as the third largest age group. Ranges and descriptive demographic variables are depicted in Table 1.

**Figure 2:**
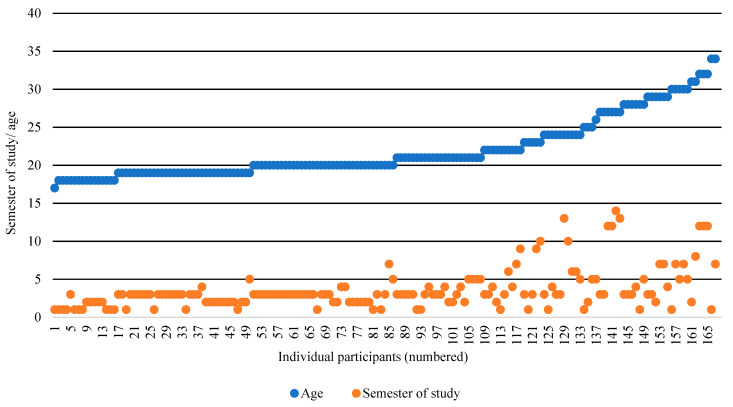
Course attendees’ distribution according to age and semester of study.

With regard to the course attendees’ age and semester of study, we compared the respective age and semester of study of each individual attendee (Figure 2).

### 3.2. Pre- vs. Post- vs. Post-Pre-Evaluation

As a first approach, we assessed evaluation data with regard to attendees *pre-, post*-, and *post-pre*-evaluation. Participants perceived a subjective reflected increase in competency in all 18 assessed domains within the two course concepts depicted by the comparison of *pre-* vs. *post-* evaluation data (all *p* < 0.001). We observed same differences in comparison of *post-pre*- vs. *post* assessments of self-reflected competencies (all *p* < 0.005).

In 11 out of 18 domains, statistically significant differences were depicted for the *pre-* vs. *post*-*pre*-data, revealing that attendees retrospectively adjusted their self-reflected pre-competencies. Within the first aid course, these differences were observed for the items emergency call, use of AED, burning, and complex first aid (all *p* < 0.050). With regard to the physical examination course, a significant change in the self-assessment of pre-course competencies was observed in all examination items (all *p* < 0.050; Table 2).

**Figure 3:**
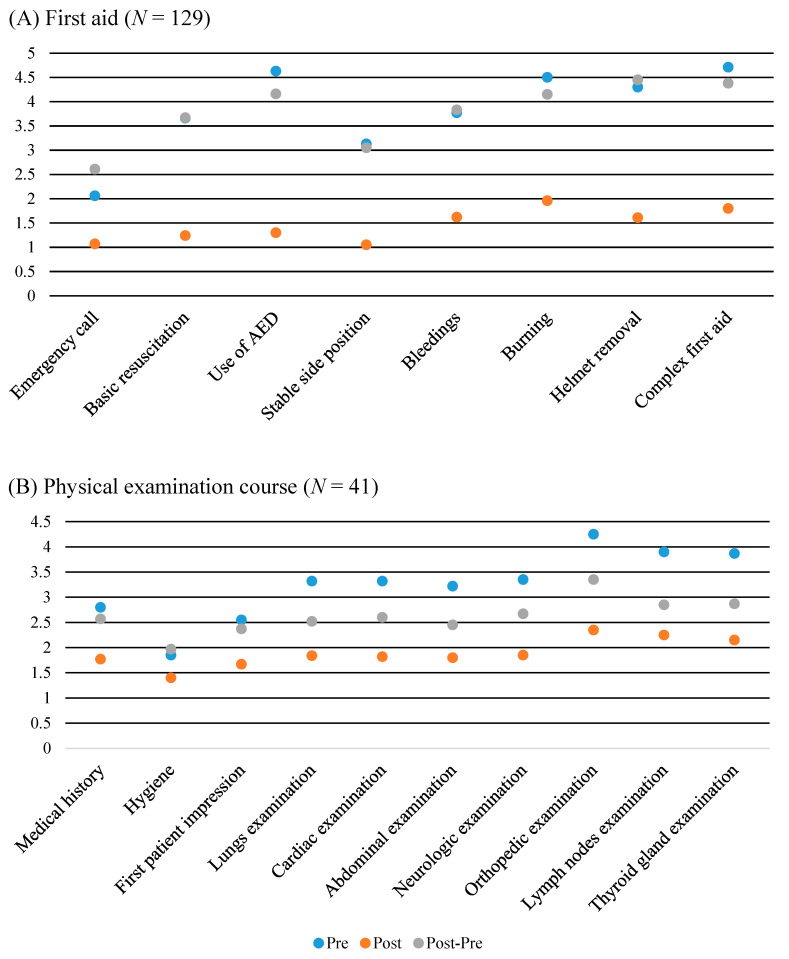
Illustrated comparisons of pre-, post-, and post-pre-evaluations in the first aid (**A**) and physical examination course (**B**).

To compare divergences between the pre-, post-, and post-pre-evaluation, we illustrated graphs with regard to each individual evaluation item and the specific course concept (Figure 3).

### 3.3. Interaction Effect between Age and Semester of Study on Concordant Self-Assessment of Competencies in the First Aid and Physical Examination Course

To identify potential influences on the self-assessment of students’ competencies, we then set out to calculate a moderated regression analysis and assess the influence of age and semester of study. Furthermore, this analysis aimed to illustrate interaction effects between these two variables on adjustments and the concordant self-reflection. As a first approach, we assessed data coming from the first aid courses. The concordant self-reflection is depicted as the difference of the *post-pre-* minus *pre-* assessment.

Negative scores therewith report that participants revealed a more negative (critical) assessment of their own competencies before the course, and afterwards retrospectively adjusted to better self-assessed competencies. Higher positive values reveal that participants actually provided better self-assessed competencies before the course than they retrospectively assessed.

We identified that the interaction of the variables age and semester of study explained a significant part of variance (∆*R*^2^ = 0.008, ∆*F* (1;1020) = 8.53, *p <* 0.005), resulting in an overall explanation of 1.3% within the concordant self-reflections variance (*F* (X; X) = 4.48, *p <* 0.005).

**Figure 4:**
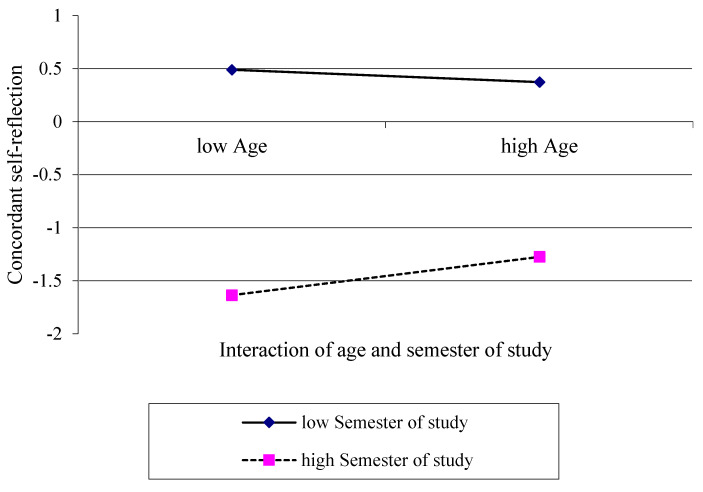
Interaction effect of age and semester of study on the concordant self-reflection in the first aid course concept illustrated as simple slopes.

For illustrating the revealed interaction effect, a simple slope analysis was calculated (Figure 4). Although the age (*R*^2^ = 0.000, *F* (1;1022) = 0.173, *p* = 0.678) did not, we identified that the semester of study (∆*R*^2^ = 0.005, ∆*F* (1;1021) = 4.690, *p* < 0.050) explained a significant amount of variance in the concordant self-assessment of competencies. Both slopes for low (*b* = −0.02, *t* = 0.17, *p* = 0.867) and high semester of study (*b* = 0.06, *t* = 0.50, *p* = 0.614) were not significantly different from zero.

Next, we analyzed the same interaction effects for evaluation data coming from the physical examination course. Depicting the moderated regression analysis revealed hereby that the interaction of age and semester of study explained a significant amount of variance on the concordant self-reflection (∆*R*^2^ = 0.03, ∆*F* (1;398) = 10.72, *p* < 0.001), resulting in an overall explanation of 3.8% within the concordant self-reflections variance (*F* (X; X) = 5.22, *p* < 0.005).

**Figure 5:**
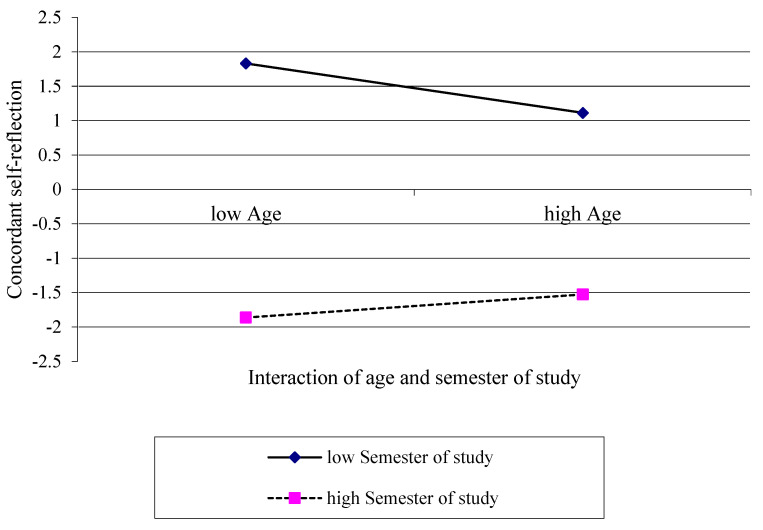
Interaction effect of age and semester of study on the concordant self-reflection in the physical examination course concept illustrated as simple slopes.

Age (*R*^2^ = 0.006, *F* (1;398) = 2.42, *p* < 0.120) and semester of study itself (∆*R*^2^ = 0.006, ∆*F* (1;397) = 2.38, *p* < 0.124) did not explain a significant amount of variance. A simple slope analysis was calculated to illustrate the revealed interaction effect (Figure 5). Slopes for low semester of study (*b* = −0.09, *t* = 1.19, *p* < 0.235) and high semester of study (*b* = 0.04, *t* = 0.65, *p* < 0.518) were not significantly different from zero.

## 4. Discussion

Within our investigation, we conducted a moderated regression analysis to quantify the interacting effects of age and semester of study on the concordant self-reflection of competencies depicted in evaluation data originating from PAL skills labs courses.

With regard to the overall cohort of *N* = 169 attendees in first aid and physical examination courses, all assessed pre- and post-comparisons revealed that participants documented a significant increase in self-determined competencies after participating. These findings are congruent with earlier investigations of smaller cohorts and other course concepts documenting perceived competency increases immediately after course-participation as part of quality assessments [23,24]. Remarkably, in 11 out of 18 assessed items, attendees significantly estimated their own competencies before the course retrospectively (*post-pre*) as different than before (*pre*). A retrospective adjustment of self-regarded competencies in training of medical skills was investigated earlier, describing how post-graduate attendees with low experiences changed their correction to lower retrospectively assessed pre-course levels [25]. In order to investigate whether there are explanations for corrective behaviors based on demographic- or study-specific aspects of participants, we conducted the presented analyses to quantify an influence of semester of study and age, as well as those variables’ interaction. Of note, in both course concepts, the interaction of the variables age and semester of study explained a significant part of the concordant self-reflections’ variance. Additionally, within the first aid course, age of participants explained a significant amount of variance. One might thereafter hypothesize that age alone may have an impact, since earlier research investigated increased age influencing personality and emotional regulations [26]. However, this was not supported by data coming from the physical examination course, revealing no significant explanation by age or semester of study alone.

Regarding both investigations, the additionally calculated simple slope analysis proved to be helpful for illustrating effects, revealing that participants with a comparatively low semester of study on trend produced better self-assessed competencies evaluations before the course than they retrospectively assessed as their pre-course levels. In contrast, higher-educated study participants in higher semesters tended to a more critical assessment of own competencies before the course and retrospectively adjusted to better pre-course competencies. One might hypothesize that participants were influenced by their prior individual educational process and evoked a memory of practical skills or trainings that they conducted earlier during their course of studies, but which they had not trained or reflected explicitly. It is reasonable that the educational process and earlier training path differed between older and younger students as well as different semesters, constituting statistically the interaction effects of the presented data here. This hypothesis might explain that pre- vs. post-pre-assessments differentiated to different directions with regard to specific evaluation items.

Of note, especially in the physical examination course, participants revealed better *post-pre*-assessments, which might be caused by the older and more trained students if compared to participants in the first aid course. Participants potentially remembered and recognized skills which they have not been explicitly trained in via the course of study due to the participation and reflection in the course. Another explanation might be a more critical attitude towards their own competence assessments educated through the earlier course of studies. This hypothesis assuming a change of self-assessment and self-confidence over time in medical students and young physicians with regard to medical trainings has likewise been raised by other researchers [27]. However, and partly controversially, in educated clinicians, Rezaiefar et al. investigated in a smaller cohort that the self-assessment of competencies was not more accurate in experienced health professionals versus early postgraduate trainees [28].

Moreover, in participants with comparative higher ages, simple slope analysis graphs approach more to the zero line, indicating more concordant self-assessments. However, slopes were not significantly different from zero; the concordant self-assessment in older students partly remains speculative.

As a limitation, the overall explanation on the concordant variance explained by the interaction of age and semester of study investigated in our statistical model was comparatively low (1.3–3.8%) and allows the assumption that other factors might have had an undetected influence. Since earlier research investigated further factors such as sex having an impact on the self-assessment of students’ practical skills, we depicted that more than 65% of the course attendees were female [29]. Research approaches in the future should implement standardized questionnaires and rating scales helping to determine further influencing factors on self-assessment and self-perception, as earlier implemented in other education research, or at least inquire participants to give an estimation of their own self-reflection skills [30]. Additionally, an analysis in other course concepts and larger cohorts might help to determine further influencing factors.

As a conclusion, we quantified that the interaction of age and semester of study has a significant influence on the concordant self-reflection of competencies. This has implications on the curriculum development, aligning with former studies postulating that an exposure to self-reflection as a metacognitive process should be introduced early in medical schools to train competencies in health professionals [31]. With the presented approach here, we feel confident that participants were encouraged to initiate self-reflective behaviors. This might be helpful for the educational process in other curricular teaching concepts and to improve self-reflection as an important non-technical skill. However, it remains partly unsolved if the accurate self-assessment and -reflection has an impact on the learning process. Therefore, prospective studies with competency-based assessments are necessary to validate findings.

## Figures and Tables

**Table 1:** Descriptive demographics of course attendees.

Domain/Variable	M	SD	Range
Age (y) (*n* = 168)	22.00	3.91	17–34
Semester of study (*n* = 168)	3.57	2.68	1–14
Earlier professional training (*n* = 165)	Yes 34	No 131	% Yes 20.6
Earlier participated in Skills Labs course (*n* = 169)	116	53	68.6
Sex (*n* = 169)	Female 111	Male 58	% Female 65.7

**Table 2:** Compared evaluation data of course attendees with regard to self-reflection of competencies.

Domain/Item	*Pre-* (1)	*Post* (2)	*Post-Pre-* (3)	*p* (1) vs. *p* (2)			*p* (1) vs. *p* (3)			*p* (2) vs. *p* (3)		
First Aid (*N* = 129)				*p*	*F*	Part. Eta2	*p*	*F*	Part. Eta2	*p*	*F*	Part. Eta2
Emergency call	2.06 (1.13)	1.07 (0.26)	2.61 (2.12)	<0.001	100.98	0.441	0.006	7.81	0.058	<0.001	74.14	0.367
Basic resuscitation	3.66 (1.44)	1.24 (0.46)	3.67 (1.33)	<0.001	361.22	0.738	0.950	0.004	0.001	<0.001	423.48	0.768
Use of AED	4.63 (1.36)	1.30 (0.47)	4.16 (1.52)	<0.001	844.16	0.868	<0.005	12.17	0.087	<0.001	462.34	0.785
Stable side position	3.13 (1.28)	1.05 (0.23)	3.05 (1.46)	<0.001	322.15	0.716	0.532	0.392	0.003	<0.001	239.30	0.652
Bleedings	3.77 (1.26)	1.62 (0.62)	3.83 (1.33)	<0.001	353.55	0.734	0.678	0.173	0.001	<0.001	336.73	0.725
Burning	4.50 (1.28)	1.96 (0.72)	4.15 (1.41)	<0.001	473.46	0.787	0.012	6.45	0.048	<0.001	371.11	0.744
Helmet removal	4.30 (1.30)	1.61 (0.72)	4.45 (1.54)	<0.001	520.00	0.804	0.305	1.06	0.008	<0.001	434.97	0.773
Complex first aid	4.71 (1.10)	1.80 (0.69)	4.38 (1.36)	<0.001	750.80	0.855	0.007	7.54	0.056	<0.001	438.27	0.774
**Physical Examination Course (*N* = 41)**												
Medical history	2.80 (1.23)	1.77 (0.76)	2.57 (1.30)	<0.001	59.46	0.598	0.226	1.51	0.36	<0.001	20.82	0.342
Hygiene	1.85 (0.76)	1.40 (0.49)	1.97 (0.85)	<0.001	20.89	0.343	0.364	0.844	0.021	<0.001	24.90	0.384
First patient impression	2.55 (0.92)	1.67 (0.65)	2.37 (1.11)	<0.001	41.34	0.508	0.301	1.10	0.027	<0.001	19.89	0.332
Lungs examination	3.32 (1.29)	1.84 (0.77)	2.52 (1.18)	<0.001	66.92	0.626	<0.001	14.90	0.272	<0.001	18.19	0.313
Cardiac examination	3.32 (1.35)	1.82 (0.80)	2.60 (1.36)	<0.001	57.66	0.590	0.006	8.45	0.174	<0.001	19.32	0.326
Abdominal examination	3.22 (1.33)	1.80 (0.87)	2.45 (1.14)	<0.001	57.64	0.590	<0.001	17.29	0.302	<0.001	20.93	0.344
Neurologic examination	3.35 (1.42)	1.85 (0.82)	2.67 (1.25)	<0.001	63.62	0.614	<0.005	9.25	0.188	<0.001	23.26	0.369
Orthopedic examination	4.25 (1.41)	2.35 (0.96)	3.35 (1.56)	<0.001	106.48	0.727	<0.001	15.16	0.275	<0.001	24.12	0.376
Lymph nodes examination	3.90 (1.39)	2.25 (1.11)	2.85 (1.44)	<0.001	66.54	0.625	<0.001	17.07	0.199	<0.005	10.62	0.210
Thyroid gland examination	3.87 (1.30)	2.15 (1.04)	2.87 (1.45)	<0.001	67.80	0.629	<0.001	19.07	0.323	<0.001	14.37	0.264

## Data Availability

The data that support the findings of this study are available from the corresponding author upon reasonable request. The data are not publicly available.
